# Antibiotic Resistance Profile, Biofilm Formation Ability, and Virulence Factors Analysis of Three Staphylococcus spp. Isolates From Urine

**DOI:** 10.7759/cureus.37877

**Published:** 2023-04-20

**Authors:** Ulrich Joël Tsopmene, Yves Somo Iwewe, Isaac Mboh Eyong, Borel Ndezo Bisso, Jean Paul Dzoyem

**Affiliations:** 1 Department of Biochemistry, University of Dschang, Dschang, CMR; 2 Department of Biomedical Sciences, University of Ngaoundéré, Ngaoundéré, CMR; 3 Department of Obstetrics and Gynecology, University of Buea, Buea, CMR

**Keywords:** biofilm formation, virulence factors, antibiotic resistance, staphylococcus, urinary tract infections

## Abstract

Background

*Staphylococcus spp*. is one of the most causative agents of urinary tract infections (UTIs). This study aimed to investigate the antibiotic resistance profile and the virulence factors, including the biofilm formation ability of *Staphylococcus spp.* isolates from urine.

Methodology

The agar disk diffusion method was used to test the susceptibility of *Staphylococcus *isolates to ten antibiotics. The biofilm formation ability was determined using the safranin microplate-based method, and the phospholipase, esterase, and hemolysin activities were assessed by the agar plate method.

Results

During the study period, a prevalence of 18.12% of urinary tract infections caused by the identified Staphylococci was obtained. All the isolated *Staphylococcus aureus* and *S. epidermidis* were resistant to cefazolin. Multi-drug resistance (MDR) was recorded in 80.01%, 81.49%, and 76.20% of *S. aureus*, *S. epidermidis,* and *S. saprophyticus* isolates, respectively. Most of the isolates were moderate biofilm formers, while 44.44%, 31.75%, and 30.16% were positive for phospholipase, esterase, and hemolysin activities, respectively. No relevant correlations were observed between the ability of biofilm formation and the resistance to antibiotics or the expression of virulence factors investigated.

Conclusion

This study shows that *Staphylococcus spp. *isolates from patients with clinical manifestations of UTIs expressed a high degree of virulence factors, including the ability of biofilm formation, and exhibited multi-drug resistance to the majority of antimicrobials commonly used for the treatment of Staphylococcal infections.

## Introduction

Urinary tract infections (UTIs) are the second most important cause of morbidity and healthcare spending, affecting people of all ages [1]. UTIs refer to the presence of microorganisms in the urinary tract, including the urinary bladder, prostate, collecting system, or kidney. Worldwide, about 150 million people are diagnosed with UTIs each year, costing the global economy over 6 billion US dollars [2]. They are principally caused by Gram-negative bacteria, but Gram-positives have emerged as important causative agents of UTIs, often associated with co-morbidities [3]. Talking about Gram-positive uropathogens, the most frequently encountered UTIs include coagulase-negative Staphylococci (with *Staphylococcus saprophyticus*, *S. epidermidis*) and coagulase-positive Staphylococci (with *S. aureus*), which are reported in the recent study in UTIs [4]. Around 10-20% of *S. saprophyticus* and 0.5-13% of *S. aureus* are associated with UTIs [3,5]. Also, *S. epidermidis* is mostly implicated in UTIs in children. However, *S. saprophyticus* is more commonly seen in young women and elderly men [6]. *S. epidermidis* in UTIs is not always benign [7]. For their pathogenicity, Staphylococci uropathogens express a plethora of virulence factors that increase their affinity for the epithelial cells of the urinary tract, allowing for their survival. These virulence factors include extracellular toxins (hemolysin), enzymes (proteases, lipases, phospholipases, and coagulases), and surface proteins (clumping factors and adhesions). Precisely, adhesin production by uropathogenic bacteria involves its adherence and invasion of host cells, toxins affecting host cells, and iron acquisition systems for Staphylococcus growth. The lipase production hydrolyzes triacylglycerol into free fatty acids and glycerol of the uroepithelial membrane [8]. The phospholipase and esterase expression enzymes help uropathogens to colonize tissues and/or obtain substrates from the host, thus promoting bacterial survival or invasion [9]. Additionally, hemolysins, proteases, and coagulases disrupt and sequester iron from host tissues or cells; iron is one critical factor that involves infection [10]. The biofilm formation by *Staphylococcus spp.* is a serious mechanism that increases antimicrobial resistance because of the barrier formed by the matrix of the biofilm. This barrier reduced the penetration and/or diffusion of antibiotics and increased the high rate of multi-resistant phenotypes [11]. The biofilm is composed of organized bacterial cell communities containing an extracellular matrix that mediates adherence to biotic and abiotic surfaces [5]. The effective management of patients suffering from bacterial UTIs commonly relies on the identification of the organisms that caused the disease and the selection of an effective antibiotic agent for the treatment [12]. Non-complicated UTIs can be treated with short courses of antibiotics, while complicated UTIs require longer and more intensive courses of antibiotics. However, extensive and inappropriate use of antimicrobial agents has invariably resulted in the development of antibiotic resistance, which, in recent years, has become a major problem worldwide [12]. This resistance to antibiotic treatments and the capacity of uropathogens to form biofilms during infections contribute to morbidity and socio-economic distress [11]. Previous studies demonstrate that the biofilm production ability of multi-drug resistance (MDR) or extensively drug resistance (XDR) strains enhances the overall resistance, potentially resulting in treatment failure [13].

The simultaneous coexistence of both virulence determinants and multiple-drug resistance can have an incremental effect on the outcome of the disease [14]. The distribution of virulence factors and their bacterial characteristics among UTIs are crucial to the understanding of UTIs' pathogenesis, which would guide clinical decision-making [15]. Therefore, this study aimed to investigate antibiotic resistance and determine the virulence factors of *Staphylococcus spp.* isolated from urine patients in the Dschang District Hospital of Cameroon.

## Materials and methods

Sampling

A cross-sectional study was carried out over a period of five months, from March to July 2021, to collect urine samples from patients attending the Dschang District Hospital. The study protocol was approved by the Institutional Ethics Committee for Research for Human Health of the University of Douala (CEI-UDo) (3128CEI-UDo/02/2021/T), Douala, Cameroon. The data and sample were collected only after informed consent was obtained from each participant.

Isolation and identification of isolates strains

Urine samples were collected by standard procedures and inoculated onto CHROMagar™ Orientation (CHROMagar, Paris, France) and mannitol salt agar. CHROMagar™ Orientation is a non-selective chromogenic culture medium intended for use in the qualitative direct detection, differentiation, and presumptive identification of uropathogens, where *S. aureus* appears golden yellow, *S. epidermidis* is colorless, and *S. saprophyticus* appears in pink color. The confirmation of the identification of *Staphylococcus spp.* isolates was done based on the standard biochemical characteristics using the API 20E test system (Biomereiux, France).

Antimicrobial susceptibility testing

The antimicrobial susceptibility of isolates was investigated using the disk diffusion method on Muller Hinton agar medium (MHA) according to the Clinical Laboratory Standard Institute (CLSI) and European Committee on Antimicrobial Susceptibility Testing (EUCAST) recommendations [16,17]. Ten antibiotics (CONDA pronadisa, France) were tested, including cefazolin (CZ, 30 µg), amikacin (AK, 30 µg), cefixime (CFM, 30 µg), kanamycin (K, 30 µg), doxycycline (DO, 30 µg), levofloxacin (LEV, 15 µg), ciprofloxacin (CIP, 30 µg), vancomycin (VA, 30 µg), erythromycin (E, 30 µg), and amikacin (AK, 30 µg). The interpretation of the categories of susceptible, intermediate, or resistant was based on EUCAST and CLSI breakpoints. Resistant isolates were further classified as single-drug resistant (SDR), double-drug resistant (DDR), and multi-drug resistant (MDR) as previously described [18]. A reference strain of *S. aureus* (ATCC BAA1026) was included in the study as a control for all the experiments performed.

Biofilm formation assay

Klrmusaoaylu and Kaşlkçl [19] described a 96-well microtiter plate assay for determining the bacterial capacity to form biofilm. Briefly, 100 µL of Mueller-Hinton broth (MHB) supplemented with 1% glucose and 100 µL of bacteria inoculum (0.75× CFU/mL) were introduced into each well of a microtiter plate and incubated at 37 °C for 24 hours. After three washes with 200 µL of phosphate-buffered saline (PBS) at a pH of 7.4 (to eliminate the free bacteria) and a 20-min fixation step with 180 µL methanol (for fixation of sessile organisms), the well was stained with 150 µL 0.5% (v/v) safranin (CV) for 15 min and washed gently three times with 200 µL PBS. The formed biofilms were then dissolved in 150 µL 95% (v/v) ethanol for 30 min. Sterile MHB 1% was used as the negative control. The biofilm formation was measured at 570 nm optical density using a microplate reader (VERSA-max, USA). From the obtained optical density value, isolates were classified by their biofilm formation capacity as non-biofilm producers, low biofilm producers, moderate biofilm producers, or strong biofilm producers according to our previously described cut-off [20].

Phospholipase activity

As described by Deorukhar and Siani [21], *Staphylococcus spp.* isolates were screened for phospholipase production by measuring the size of the zone of precipitation after growth on egg yolk agar medium. The egg yolk agar medium was prepared by dissolving 38 g of Muller-Hinton agar (MHA), 24.4 g of NaCl, 2.5 g of CaCl_2_, and 10% of sterile egg yolks in 440 mL of distilled water and sterilizing for 15 minutes at 121 °C. The culture medium was then cooled to about 45-50 °C, followed by the addition of 2 mL of the supernatant of an initially centrifuged 8% egg yolk at 5000 g for 30 minutes. The prepared culture medium was then poured into Petri dishes and allowed to solidify. Then, 10 µL of bacterial suspension was added to the sterile egg yolk MHA and incubated at 37 °C for five days. After incubation, colony diameter and colony diameter plus the surrounding precipitation zone were measured (in mm), and phospholipase activities (Pz) were calculated as follows:

Pz = Diameter of the colony/Diameter of the colony + Precipitation zone

Phospholipase activity was interpreted as follows: negative for Pz=1 and positive Pz<1

Esterase activity

Esterase activity was measured using the Tween 80 opacity test medium [22], which was prepared with 10 g of bacteriological peptone, 5 g of sodium chloride, 0.1 g of calcium chloride, 15 g of agar, and 1000 mL of distilled water. After the medium was autoclaved, 10 µL of bacterial isolate suspension was inoculated as a spot and incubated at 37 °C for 10 days. The formation of a precipitation zone around colonies was indicative of positive esterase activity.

Hemolytic activity was evaluated by blood plate assay, according to the previously described method [23]. Sheep blood MHA was prepared by adding 7 mL of fresh sheep blood to 100 mL of MHA supplemented with 3% glucose. Suspensions equal to 0.5 McFarland turbidity from the pure culture of the bacteria colonies were prepared. Ten microliters of this suspension were spotted on prepared media and incubated at 37 °C for 48 hours. The presence of a distinctive translucent halo or precipitation zone around the deposition site indicated positive hemolytic activity.

Statistical analysis

A Pearson correlation test was used to evaluate the relationship between the biofilm formation ability and the antimicrobial susceptibility or virulence factor activities. Data analyses were performed using GraphPad Prism 8.0. All tests were considered significant at p ˂ 0.05.

## Results

Prevalence of identified *Staphylococcus spp.* infection in the studied population

A total of 62 isolates of *Staphylococcus spp.* were identified from 342 urine samples collected from patients with clinical manifestations of UTI attending the district hospital of Dschang, resulting in a prevalence of 18.12%. *S. epidermidis* was most predominant (43.54%, 27/62) followed by *S. saprophyticus* (33.87%, 21/62) and then *S. aureus* (22.58%, 15/62).

Susceptibility to antibiotics and resistance profile

**Figure 1:**
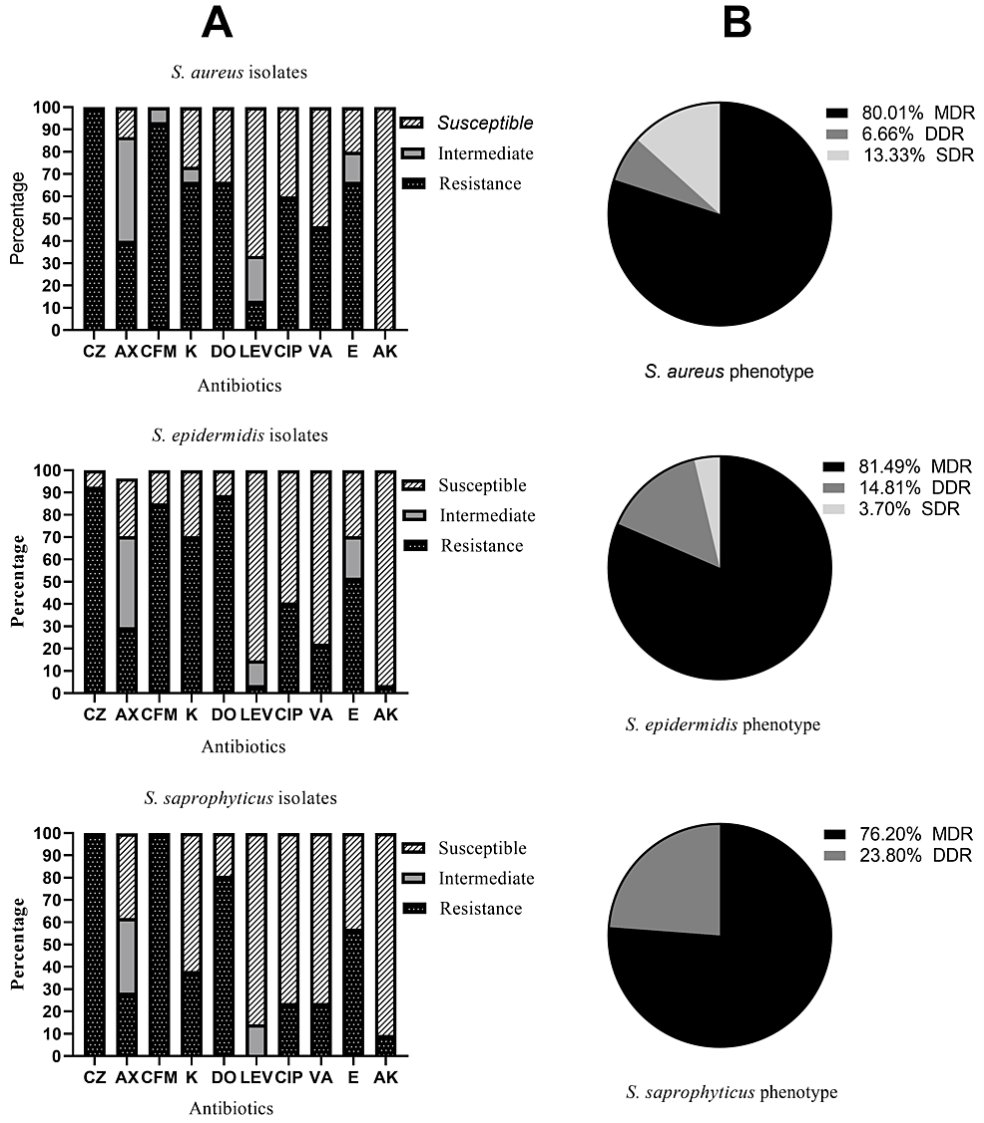
Antibiotic susceptibility (A) and resistance profile (B) of Staphylococcus spp. CZ: cefazolin, K: kanamycin, AX: amikacin, CFM: cefixim, DO: doxycycline, LEV: levofloxacin, CIP: ciprofloxacin, VA: vancomycin, E: erythromycin, AK: amikacin, MDR: multi-drug resistant, DDR: double-drug resistance, SDR: single-drug resistance.

The antibiotic susceptibility of *Staphylococcus spp.* was evaluated, and results are presented in Figure 1A. It was observed that all of the *S. aureus* isolates were resistant to cefazolin 100% (15/15), cefixime 93.33% (14/15), erythromycin 66.66% (10/15), kanamycin 66.66% (10/15) and doxycycline 60% (9/15). *S. epidermidis* showed high resistance to cefazolin 92.59% (22/27), doxycycline 88.88% (24/27), cefixime 85.18% (23/27), and kanamycin 70.37% (19/27). Similarly, all strains of *S. saprophyticus* showed resistance to cefazolin 100% (21/21) and cefixime 100% (21/21), followed by doxycycline 80.95% (17/21). Almost all the tested isolates were susceptible to amikacin. *Staphylococcus spp.* Were also classified according to their resistance phenotype (Figure 1B). Results revealed that all the isolates showed the multidrug resistance phenotype (MDR). Among the 63 isolates tested, 79.36% (50/63) were classified as MDR. among which, 81.49% of isolates of *S. epidermidis* (22/27), 80% of *S. aureus* isolates (12/15), and 76.20% of *S. saprophyticus* isolates (16/21).

Biofilm formation by *Staphylococcus spp*.

**Figure 2:**
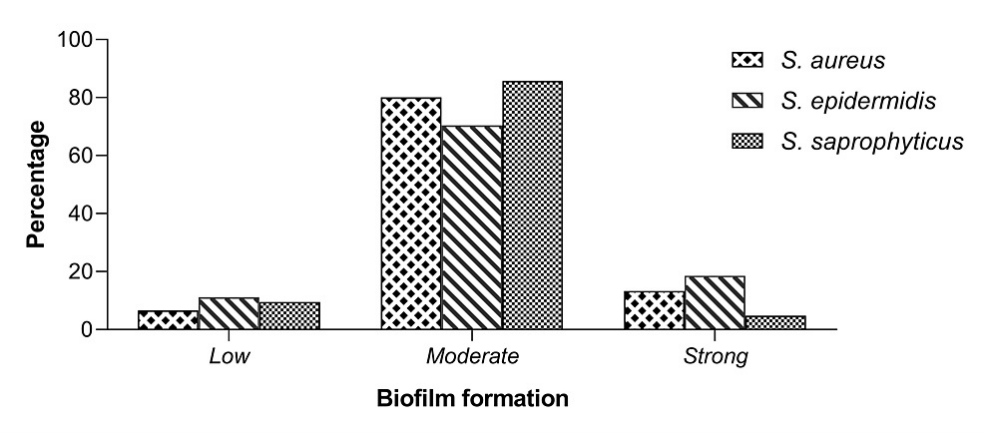
Biofilm formation by Staphylococcus spp.

The biofilm formation test revealed that 100% of our isolates were biofilm formers (Figure 2). Among 63 isolates of Staphylococci, 12.69% were strong biofilm producers (8/63), 77.77% were moderate biofilm producers (49/63), and 9.52% were low biofilm producers (6/63).

Hydrolytic enzyme activities among the *Staphylococcus spp.* isolates

*Staphylococcus spp.* isolates were screened for the presence of four different hydrolytic enzyme activities, namely phospholipase, esterase, and hemolysin activities (Table 1). As shown in Table 1, phospholipase activity was found to be the most common hydrolytic activity detected in a maximum of 28 (44.44%) isolates, followed by esterase activity detected in 20 (31.74%) of the isolates, very close to the hemolytic activity found in 19 (30.15%) isolates. Among the 15 coagulase-positive *S. aureus* isolates, 14 (93.33%) were found positive for phospholipase activity. However, esterase activity was the least frequently detected among the *S. saprophyticus* isolates, being produced by only one (4.76%) out of 21 isolates.

**Table 1:** Phospholipase, esterase, and hemolytic activity expression in Staphylococcus spp. isolates. Pos: positive; Neg: negative; No: number

Staphylococcus species	No. of isolates	Phospholipase no (%)	Esterase no (%)	Hemolysin No (%)
S. aureus	15	14 (93.33)	8 (53.33)	7 (46.66)
S. epidermidis	27	7 (25.92)	11 (40.74)	6 (22.22)
S. saprophyticus	21	7 (33.33)	1 (4.76)	6(28.57)
Total	63	28 (44.44)	20 (31.74)	19 (30.15)

Correlation

The correlation between the resistance of *Staphylococcus spp.* and biofilm formation is presented in Table 2. Except for amikacin, for which the resistance of *S. saprophyticus* isolates was found to be correlated with biofilm formation (r = −0.44 and p = 0.04). The correlations between the phenotypic expression of biofilm formation and the activity of the hydrolytic enzymes presented in Table 2 show that biofilm formation had a significant negative correlation with the expression of esterase activity in *S. epidermidis* isolates (r = −0.38 and p = 0.04). In all other cases, biofilm formation did not show a significant correlation with resistance to the investigated antibiotics or virulence factors. No significant correlation was observed between the biofilm formation of isolates and their resistance to the investigated antibiotics.

**Table 2:** Correlation matrix between the biofilm formation ability, antibiotics resistance, and other virulence factors in Staphylococcus spp. isolates. S. a: *Staphylococcus aureus*; S. e: *Staphylococcus epidermidis*; S. s: *Staphylococcus saprophyticus*; CZ: cefazolin; AX: amikacin; CFM: cefixime; K: kanamycin; DO: doxycycline; LEV: levofloxacin; CIP: ciprofloxacin; VA: vancomycin; E: erythromycin; AK: amikacin; PPL: phospholipase; EST: esterase; HML: hemolysin; r: Pearson correlation coefficient; p: statistics.

Biofilm formation	Isolates		Antibiotics										Virulence factors		
			CZ	AX	CFM	K	DO	LEV	CIP	VA	E	AK	PPL	EST	HML
	S. a	r	−0.49	−0.25	0.26	−0.03	0.12	0.29	−0.24	0.11	0.00	−0.22	0.05	0.27	−0.13
		p	0.06	0.35	0.33	0.89	0.66	0.29	0.38	0.69	0.98	0.42	0.84	0.31	0.63
	S. e	r	−0.01	−0.21	0.13	−0.01	−0.23	−0.17	−0.14	−0.15	0.05	−0.11	−0.38	−0.22	−0.18
		p	0.94	0.28	0.51	0.93	0.24	0.39	0.46	0.44	0.79	0.57	0.04	0.25	0.35
	S. s	r	−0.29	−0.23	0.27	−0.24	−0.35	−0.15	−0.28	−0.20	−0.35	−0.44	0.04	0.03	−0.21
		p	0.19	0.31	0.22	0.27	0.11	0.51	0.21	0.37	0.11	0.04	0.83	0.86	0.35

## Discussion

Urinary tract infection is a very frequent infection in hospital patients and the community, and the increasing issue of antimicrobial resistance in the community setting and infections with Staphylococci pathogens have become a prevalent problem among UTI patients. This study revealed the presence of three *Staphylococcus spp.* (*S. aureus*, *S. saprophyticus*, and *S. epidermidis*) in urine samples. The isolation of *S. aureus* from UTIs is well documented in the literature [3]. However, *S. epidermidis* has usually been considered a urinary contaminant; this finding indicates that caution should be taken before making this assumption [7]. Effective management of patients with bacterial UTIs generally depends on the identification of aetiologic organisms and the choice of effective antibiotics. The results of the antibiotic susceptibility profiles of the Staphylococci isolates in the urine samples showed varied percentages of sensitivity and resistance. The *S. aureus* strain isolated in this study showed a very high level of resistance to cefazolin (100%) and cefixime (93.33%). On the other hand, the relatively low resistance rate of *S. aureus* was observed with amikacin and levofloxacin. Similarly, previous studies had also reported the high resistance of *S. aureus* against cefixime (80%) [24]. It is noteworthy that all 15 *S. aureus* isolates tested were sensitive to amikacin, whereas only 13.33% were resistant to levofloxacin. The lack of resistance of *S. aureus* to amikacin obtained in our study is different from the high resistance profile reported in previous studies [24]. Therefore, according to the observed results, amikacin and levofloxacin could be alternative molecules in the treatment of UTIs caused by *S. aureus* in the studied population. Resistant *S. epidermidis* strains are considered to be one of the major causes of human clinical infections in hospitals. Our study showed a high resistance of *S. epidermidis* to cefazolin, followed by doxycycline, and then cefixime. Regarding antibiotic families, our studies showed that *S. epidermidis* had a lower resistance to amikacin, which belongs to the penicillin family, compared to vancomycin, a quinolone. *S. saprophyticus* is the most frequent Gram-positive bacteria in UTIs. It shows the highest prevalence of resistance was observed with two cephalosporins, cefazolin (100%) and cefixime (100%), followed by kanamycin (80.95%). Comparatively, the previous work reported a lower rate of resistance of *S. saprophyticus* to antibiotics: cefixime (90%), cefazolin (38%), and kanamycin (64%) [24]. Studies of the antimicrobial resistance profiles of *Staphylococcus spp.* are very useful and can provide data that assist with strategies to avoid the spread of these multi-drug resistant (MDR) organisms. Among the sixty-three strains of Staphylococci tested, 50 (79%) exhibited the MDR phenotype. The prevalence of the MDR phenotype obtained was 80.01%, 81.49%, and 76.20% for *S. aureus*, *S. epidermidis*, and *S. saprophyticus*, respectively.

The ability of *Staphylococcus spp.* to cause infections is related to the expression of various virulence factors such as surface proteins, biofilms, exoenzymes, exotoxins, and exfoliative toxins. Biofilm formation is the most important virulence factor that participates in pathogenicity by increasing the resistance of the constituent microbes to antibiotics and host defenses. The biofilm formation test reveals that 100% of our isolates were biofilm formers to various extents, classified as strong, moderate, and weak biofilm formers. More than 75% of *Staphylococcus spp.* fell into the moderate biofilm former group. Although no relevant correlations were found between the ability of biofilm formation and the resistance to antibiotics, the biofilm producers, *Staphylococcus spp.*, isolated in this study were resistant to multiple antibiotics, which agrees with other previous studies. The formation of the biofilm by *Staphylococcus spp.* facilitates the acquisition of the MDR phenotype and thus enhances their pathogenicity and the failures in antibiotic therapy, resulting in the persistence and recrudescence of urinary tract infections. Nearly all strains of Staphylococci secrete several extracellular enzymes for their pathogenicity, whose function is thought to be the disruption of host tissues and/or inactivation of the host antimicrobial mechanism [25]. In this study, we found that phospholipase (44.44%) was the most abundant hydrolytic enzyme in all *Staphylococcus spp.*, followed by esterase (31.74%) and hemolysin (30.15%). Virulence factors from *Staphylococcus spp.* have rarely been described for UTIs. These extracellular hydrolytic enzymes specifically favor the development of pyelonephritis, cystitis, and asymptomatic bacteriuria [26]. In this study, the three virulence factors were identified in three different *Staphylococcus spp.*, including *S. aureus*, *S. saprophyticus*, and *S. epidermidis*. We found that *S. aureus* had the highest activity of all the three hydrolytic enzymes studied: 93.33% of phospholipase, 53% of esterase, and 46.66% of hemolysin activities. This finding is consistent with the literature since a majority of studies related to the virulence factors of Staphylococcus have been conducted on *S. aureus* isolates, which are considered the most pathogenic species of Staphylococcus [27,28].

Limitations

The main limitation of the study is the limited number of samples collected and, therefore, the small number of *Staphylococcus spp.* isolates identified. Thus, a high number of samples collected over a longer period would provide a more precise prevalence value and probably a good resistance profile.

## Conclusions

This study shows that *Staphylococcus spp.* isolates from patients with clinical manifestations of UTIs expressed a high degree of virulence factors, including the ability of biofilm formation, and exhibited multi-drug resistance to the majority of antimicrobials commonly used for the treatment of Staphylococcal infections. Moreover, this study highlighted the presence of *S. epidermidis* isolates commonly considered urinary contaminants. Our findings emphasize the importance of choosing an appropriate antimicrobial regimen based on antibiotic susceptibility patterns and the need for the implementation of effective infection control practices to hinder the spread of MDR *Staphylococcus spp.* isolates.
